# Supplementary material for: The interactome of intact mitochondria by cross-linking mass spectrometry provides evidence for coexisting respiratory supercomplexes
Source: Mol Cell Proteomics. 2017 Dec 8;17(2):216–32. doi: 10.1074/mcp.RA117.000470 (PMC5795388; doi:10.1074/mcp.RA117.000470)
Supplement: Supplemental Data [file supp_RA117.000470_133922_0_supp_23978_fzffwf.zip › spectra_annotation/mito_spectra_annotation/77-1-10-1-15-1.pdf]

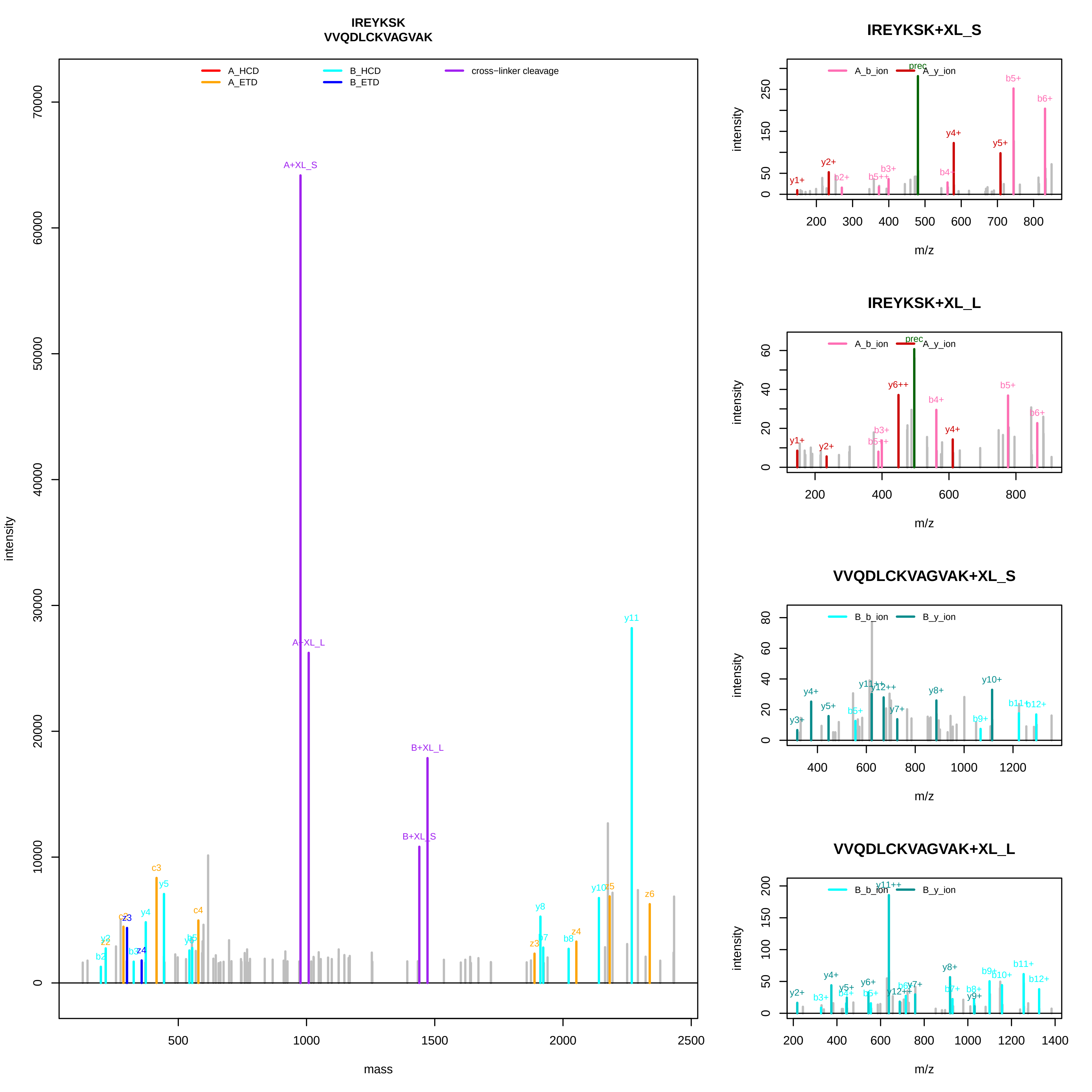
IREYKSK
VVQDLCKVAGVAK
A_HCD
A_ETD
B_HCD
B_ETD
cross−linker cleavage
intensity
mass
0
10000
20000
30000
40000
50000
60000
70000
500
1000
1500
2000
2500
A+XL_S
A+XL_L
B+XL_L
B+XL_S
y11
y10
z5
z6
c3
y5
y4
c4
c2
z3
z4
b2
y2
z2
b3
y6
b5
y8
b7
z3
b8
z4
IREYKSK+XL_S
A_b_ion
A_y_ion
prec
y1+
y2+
b2+
b5++
b3+
b4+
y4+
y5+
b5+
b6+
m/z
IREYKSK+XL_L
y6++
y4+
VVQDLCKVAGVAK+XL_S
B_b_ion
B_y_ion
y3+
y4+
y5+
b5+
y11++
y12++
y7+
y8+
y10+
b9+
b11+
b12+
VVQDLCKVAGVAK+XL_L
y11++
y2+
b3+
b4+
y6+
b6+
y7+
y12++
y8+
b7+
b8+
y9+
b9+
b10+
b11+
b12+
